# Time to achieve a patient acceptable symptom state in myasthenia gravis

**DOI:** 10.3389/fneur.2023.1187189

**Published:** 2023-06-16

**Authors:** Rodrigo Martinez-Harms, Carolina Barnett, Vera Bril

**Affiliations:** ^1^Ellen & Martin Prosserman Centre for Neuromuscular Diseases, Toronto General Hospital, Toronto, ON, Canada; ^2^Department of Medicine, University Health Network, University of Toronto, Toronto, ON, Canada

**Keywords:** myasthenia gravis, patient acceptable symptom state, myasthenia gravis impairment index, single simple question, satisfactory disease status

## Abstract

**Introduction:**

The patient acceptable symptom state (PASS) is a reliable way to characterize a patient’s satisfaction with their disease state in a “Yes”/“No” dichotomous manner. There is limited data on the time required to reach an acceptable state in Myasthenia Gravis (MG). We aimed to determine the time to reach a first PASS “Yes” response in patients at MG diagnosis and a PASS “No” status, and also to determine the influence of various factors on this time.

**Methods:**

We performed a retrospective study of patients diagnosed with myasthenia gravis who had an initial PASS “No” response and defined the time to reach a first PASS “Yes” by Kaplan–Meier analysis. Correlations were made between demographics, clinical characteristics, treatment and disease severity, using the Myasthenia Gravis Impairment Index (MGII) and Simple Single Question (SSQ).

**Results:**

In 86 patients meeting inclusion criteria, the median time to PASS “Yes” was 15  months (95% CI 11–18). Of 67 MG patients who achieved PASS “Yes,” 61 (91%), achieved it by 25  months after diagnosis. Patients who required only prednisone therapy achieved PASS “Yes” in a shorter time with a median of 5.5  months (*p* = 0.01). Very-late-onset MG patients reached PASS “Yes” status in a shorter time (HR = 1.99, 95% CI 0.26–2.63; *p* = 0.001).

**Discussion:**

Most patients reached PASS “Yes” by 25  months after diagnosis. MG patients who only required prednisone and those with very-late-onset MG reach PASS “Yes” in shorter intervals.

## Introduction

Myasthenia gravis (MG) is a chronic autoimmune disease that leads to impairment of neuromuscular transmission in skeletal muscles, most commonly caused by antibodies to the acetylcholine receptor (AChR) (1). Severity varies from purely ocular presentations of MG, with mild weakness limited to the ocular muscles causing ptosis and diplopia, to generalized MG, with generalized limb and bulbar weakness causing mild to severe dysarthria, dysphagia, and weakness of arms and legs in addition to ocular manifestations. Short and long-long term fluctuations characterize the clinical course of MG (2, 3).

In two-thirds of patients, the initial presentation is that of ocular symptoms. These patients are reported to transition to generalized myasthenia gravis in the first 2 years of diagnosis in 80% of cases. The course of generalized MG varies in time; most patients experience intermittent worsening of symptoms and typically reach the maximal severity in the first two to 3 years from disease onset (2, 4, 5).

Different risk factors may impact the clinical course of MG patients. The principal predictors in MG are the initial clinical features, age of diagnosis and AChR antibody status (6–8). The influence of thymoma is debatable as an outcome indicator (9, 10). MG scales are a reliable way to describe the severity of MG and monitor disease evolution. Patient-reported outcomes such as the Myasthenia Gravis Impairment Index (MGII) (11, 12), the single simple question (SSQ) (13), and the patient acceptable symptom state (PASS) (14) are such measures.

The PASS determines a threshold of the patient’s satisfaction with their Myasthenia Gravis status, determined by a dichotomous question of “Yes” or “No.” Achieving PASS “Yes” status is a patient-centred treatment goal. A recent study of the SSQ in MG showed a positive association of the PASS response with SSQ. An SSQ of 72.5% or higher predicted a positive PASS response (PASS “Yes”) (15). An MGII score threshold of ≤10 points has high predictive value for a PASS positive response (14). PASS thresholds and the PASS question itself can be used for assessing long-term outcomes, anchored on the patient’s assessments of their own health, rather than from a clinician’s perspective, such as is the case with the Myasthenia Gravis of America (MGFA) post-intervention status (16, 17).

MG has significant implications for both patients and the health care system. Knowing the time required to reach a first PASS “Yes” in MG may help direct the treatment approach and prognosis. The initial prognosis after MG diagnosis has a meaningful impact on the patient’s perception of their disease and can help with counseling about their condition and expected duration of impairment. There is a lack of evidence on the time to reach satisfactory disease status using patient-centred outcomes, since most studies assessing long-term outcomes have traditionally used the MGFA classification (i.e., remission, minimal manifestation status), which is rated by a clinician. The present study aimed to measure the time it takes to reach a first PASS “Yes” response from MG diagnosis in patients who are initially dissatisfied with their disease condition as characterized by a PASS “No” and to determine which factors may be associated with a shorter time to achieve satisfactory symptom control.

## Materials and methods

We performed a retrospective chart review of patients with MG who had attended the Prosserman Family Neuromuscular clinic at the University Health Network, Toronto General Hospital, between December 2016 to March 2021. The study was approved by the University Health Network Research Ethics Board.

In our clinic, every MG patient are followed typically every 3 to 6 months and answer a series of validated measures at each visit, including the PASS, SSQ and MGII scales. For this study, we included patients who were diagnosed with MG and had initial unsatisfactory disease symptoms determined by a negative response to the PASS question (PASS “No”), or a score in the MGII >10, if the PASS question was not available at the first visit. Additional inclusion criteria included: a minimum follow-up of 3 months from the date of diagnosis and patients who had completed the MGII, SSQ and PASS responses. The MG diagnosis was confirmed by clinical presentation supported by abnormal repetitive nerve stimulation (RNS) or single fiber EMG (SFEMG) and/or positive antibodies against AChR or MuSK. The included patients’ clinical presentations were only ocular or generalized MG. Patients with congenital myasthenic syndromes or patients diagnosed after their baseline visit with an alternative diagnosis were excluded. We abstracted the following data at the baseline (first clinical visit): demographics, clinical presentation, MG scales (SSQ, MGII and PASS responses), serological status, thymoma status, electrophysiologic test results and medical treatment. The data abstracted at the follow-up visits included PASS response, MGII and SSQ scores. In addition, we collected medication history during the follow-up period.

## Measures

To assess PASS, patients answered the following question: “Considering all the ways you are affected by Myasthenia, if you had to stay in your current state for the next months, would you say that your current disease status is satisfactory?,” with a dichotomous response: “Yes”/“No” (14).

The single simple question (SSQ) rates how patients perceive their disease status as a continuous variable. The SSQ is a broad disease impact scale and measures the overall disease status of the myasthenia gravis patient and can be used to assess treatment efficacy overall in longitudinal follow-up. The implementation is by asking what percent of normal patients are with respect to their overall MG status; 0% being the worst, and 100% being normal (13, 15).

The MGII is a measure of MG severity, that has demonstrated feasibility, reliability, and construct validity. It consists of 22 patient-reported and 6 examination items. Total scores for the MGII can range from 0 to 84; higher scores represent worse disease severity (12).

All statistical analyses were conducted with R statistical software and RStudio (version 2021.09.1 + 372). Clinical and demographic data are reported in terms of means and standard deviations for continuous variables or percentages for discrete variables. Kaplan–Meier analysis was used to determine the time course to reach PASS “Yes” and analysis of the cumulative proportion of patients who achieved a PASS “Yes” response each month. Patients who had an initial PASS “Yes” response were not included as they did not meet eligibility criteria for the study. Patients with PASS “No” were included until their final visit. Multivariate analyses with Kaplan–Meier and Cox regression hazard model adjusted for possible confounding factors such as age, sex, clinical presentation, antibody status and thymoma were done to correlate the time to achieve a PASS “Yes” with demographics, clinical presentation, the baseline MGII and SSQ, thymoma status, antibody status and treatment. *p* values <0.05 were considered statistically significant.

## Results

A total of 86 patients who satisfied the inclusion criteria were included. Details of the clinical and demographic data are presented for all the patients at baseline, those who achieve PASS “Yes” and those who stay in PASS “No” in Table 1.

**Table 1:** Baseline profile of MG patients.

	Total (86) Mean ± SD (range) / number (percentage)	PASS “Yes” (67) Mean ± SD (range)/number (percentage)	PASS “No” (19) Mean ± SD (range)/number (percentage)
Age of onset (years)	61.4 ± 14.9 (23–85)	61.7 ± 14.8 (23–85)	62 ± 15.4 (27–81)
Male	44 (51.2%)	36 (53.7%)	8 (42.1%)
Female	42 (48.8%)	31 (46.3%)	11 (57.9%)
Ocular MG at diagnosis	25 (29%)	21 (31.3%)	4 (21.05%)
Generalized MG at diagnosis	61 (70.1%)	46 (68.7%)	15 (78.9%)
MGII score at diagnosis	24.5 ± 12.2 (3–75)	23.1 ± 12.2 (3–75)	27 ± 11 (9–48)
SSQ at diagnosis	58.9% ± 21.9 (10–90)	60.5% ± 23.2 (10–90)	53.8% ± 15.5 (25–85)
History of Thymoma	17 (19.8%)	12 (17.9%)	5 (26.3%)
Positive antibody serology	57 (66.3%)	44 (65.7%)	13 (68.4%)
Positive RNS	28 (33.2%)	20 (29.9%)	8 (42.1%)
Positive SFEMG	81 (94.1%)	63 (94%)	18 (94.7%)

**Figure 1:**
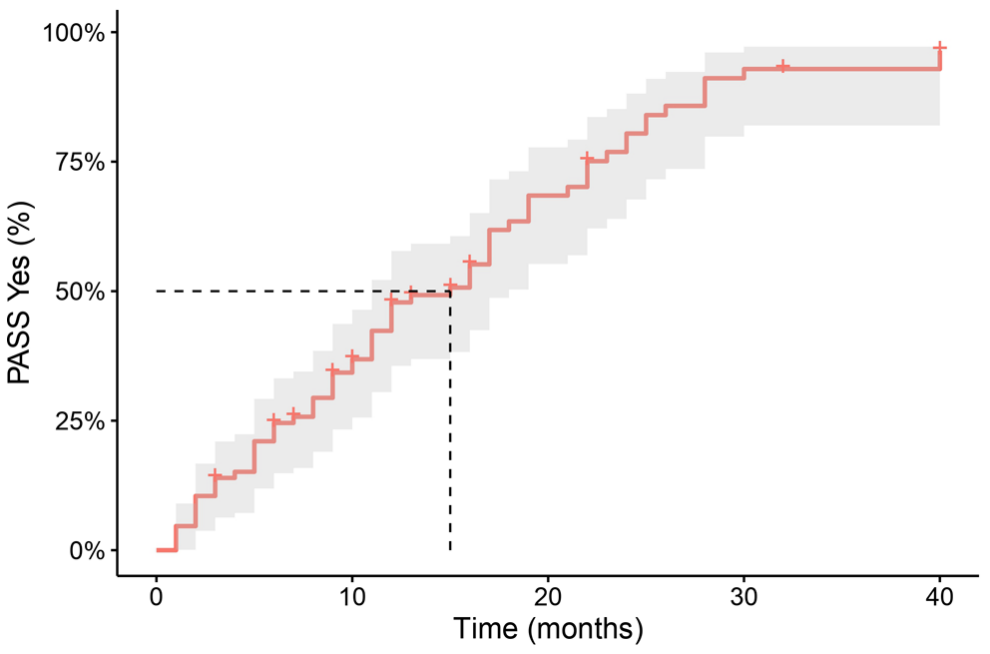
Time to PASS “Yes” with Kaplan–Meier: The median time to PASS “Yes” was 15  months (95% CI 11–18). PASS, patient acceptable symptom state.

From the total cohort, 67 (77.9%) patients reached a PASS “Yes” response during follow up, by the time of data collection. The mean time to reach PASS “Yes” was 12.8 months ±8.9 (range 1–40 months), and the median time to PASS “Yes” using the Kaplan–Meier analysis was 15 months (95% CI 11–18), as shown in Figure 1. In addition, looking at the cumulative proportion of those who reached a PASS “Yes” response, 61 (91%) achieved it by 25 months. The mean follow-up time in patients that remain as PASS “No” was 11 months (SD ± 8.9) (range 5–40 months). We did not find significant differences in the baseline clinical or demographic characteristics between patients who reached PASS “Yes” from the PASS “No” cohort or from the total population.

Very-late-onset MG (patient diagnosed at or after 65 years old) was significantly associated with a shorter time to PASS “Yes” in the cox proportional hazard model (HR = 1.99, 95% CI 0.26–2.63; *p* = 0.001). There were no significant differences in times to PASS “Yes” and sex, clinical presentation (ocular compared with generalized), thymoma status, antibody status, and abnormal electrodiagnostic tests by Kaplan–Meier and Cox regression analysis.

The clinical presentation, MGII and SSQ values at the PASS “Yes” evaluation are presented in Table 2. The mean MGII and SSQ scores at diagnosis in patients who reach PASS “Yes” were 23.1 (SD ± 12.2) and 60.5 (SD ± 23.2), respectively, similar to those with PASS “No.” At the PASS “Yes” evaluation, the mean MGII was 9.8 (SD ± 9.2) and the SSQ score 80.6 (SD ± 15.4). The MGII score of patients at the PASS “Yes” evaluation decreased significantly compared to the MGII at the diagnosis (*p* < 0.001). The SSQ percentage increased significantly from diagnosis to the PASS “Yes” evaluation (*p* < 0.005). There were no significant correlations in multivariate analyses between the severity of the MGII or SSQ scores at baseline and the time to reach PASS “Yes.” There were no significant differences in time course to achieve PASS “Yes” comparing patients with higher MGII (MGII >20) or lower SSQ (<72.5%) scores with lower MGII (MGII <20) or higher SSQ (>72.5%) scores at diagnosis of MG. In addition, at the PASS “Yes” evaluation, 4 patients (6%) had a 100% SSQ score and 8 patients (11.9%) had a score of 0 in the MGII.

**Table 2:** Scales and clinical involvement of MG patients at PASS “Yes.”

	PASS “Yes” (67) Mean ± SD (range)/number (percentage)
MGII at PASS “Yes”	9.8 (0–37 ± 9.2)
SSQ at PASS “Yes”	80.6 (35–100 ± 15.4)
Ocular MG	15 (22.4%)
Generalized MG	52 (77.6%)

In our sample, 38 patients (43.7%) were on a combination of prednisone and non-steroidal immunosuppressant therapy, 20 (23%) on prednisone alone, 16 (18.4%) on non-steroidal immunosuppressant monotherapy, and 12 (13.8%) on an acetylcholinesterase inhibitor alone. The non-steroidal immunosuppressive drugs in our cohort were azathioprine, mycophenolic acid, mycophenolate mofetil or intravenous immune globulin. Baseline severity did not influence choice of treatment (data not shown).

**Figure 2:**
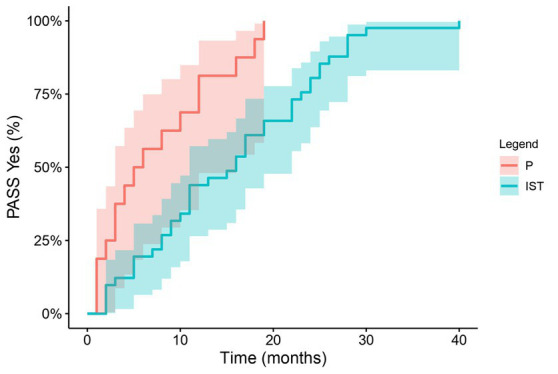
Treatment and time to PASS “Yes”: The time to PASS “Yes” with Kaplan–Meier analysis in patients only on prednisone and those who required non-steroidal immunosuppressants. PASS, patient acceptable symptom state; P, prednisone; IST, non-steroidal immunosuppressants.

The patient age did not differ significantly between the patients treated with prednisone alone (mean 66 years) and those who required non-steroidal immunosuppressants (mean 59.8 years) (*p* = 0.11). The patients who required only prednisone therapy had a significantly shorter time course to achieve a PASS “Yes” (median 5.5 months (95% CI 3–16)) compared with patients who required non-steroidal immunosuppressive treatment alone or in combination with prednisone (median 16 months (95% CI 11–22), *p* = 0.01) presented in Figure 2.

## Discussion

In this cohort, 78% of patients achieved a first PASS “Yes” status during the study period, with a median time to achieve a PASS “Yes” of 15 months. The majority of patients who achieved PASS “Yes,” did so by 25 months after diagnosis. These results are similar to previously published data on MG progression in which complete MG remission is significantly higher after the first 2 years of illness (2, 4, 5). These observations are meaningful when planning the long-term treatment of the initially diagnosed MG patient.

Patients with very-late-onset MG are 1.99 times as likely to reach PASS “Yes” sooner compared with patients under 65 years at onset, consistent with previous studies showing that older patients diagnosed with MG have a favorable long-term prognosis and good response to immunosuppressive medications (8, 18).

Generalized MG at diagnosis was the most frequent clinical presentation in 70.1% of our patients. This relatively high percentage might be due to the requirement of an initial PASS “No” response or a minimum of 10 points in the MGII score at diagnosis for inclusion in the study. Interestingly, those with generalized MG at baseline did not take a longer time to achieve a PASS “Yes.”

In contrast with other studies, we did not find associations between antibody status and patient prognosis which was unexpected, although AChR antibody status does not correlate well with clinical impairments in the individual patient and our sample may have been smaller than in other studies (6). The presence of thymoma or thymic hyperplasia did not change the time course of patients to reach a PASS “Yes” response, and this finding is supportive of earlier studies showing similar MG outcomes in those with and without thymoma (9, 19).

The PASS “Yes” response is associated with significantly lower MGII and higher SSQ measures of MG severity. This confirms the obvious assumption that patient satisfaction as indicated by a PASS “Yes” response is influenced by the disease severity. These results confirm previous data from our centre on the association of PASS with the MGII score and the SSQ scores (14, 15). In our cohort, there was no significant relationship between the baseline severity of MG on MGII and SSQ and the time required to reach a PASS “Yes.” This indicates that baseline severity does not influence the time require to reach a satisfactory disease status.

In our study, the cohort of patients who stayed on PASS “No” showed no significant differences in terms of their age of onset, sex, antibody status, or history of thymoma when compared to patients who achieved the PASS “Yes.” However, the limited number of patients in the PASS “No” group may have affected the statistical power of these results (14).

The required treatment type was a strong predictor of the time to reach a positive outcome; patients on treatment with prednisone alone had a shorter time to reach PASS “Yes,” in a median time of 5.5 months. These findings are consistent with the marked effectiveness of prednisone in the remission of MG symptoms and the relatively rapid time to response of approximately 1–3 months (3, 20). The relatively rapid onset of action and effectiveness in MG account for the ongoing widespread use of corticosteroids in MG despite the many side-effects. In contrast, patients who required non-steroidal immunosuppressive treatment had unsatisfactory symptoms for a longer interval. This could be attributed to a few factors, including the failure of the first-line treatment, the drug effectiveness, or the prolonged latency period before an effective response is observed with these agents, which can last for 6–12 months or even longer (3, 20).

The criteria for treatment selection in our clinic are diverse, considering various factors such as patient preferences, age, comorbidities, drug side effects, and tolerability. The baseline patient’s severity does not directly influence the therapy decision. Patients may start treatment with steroids, a non-steroidal immunosuppressant, or a combination of both. This treatment selection bias is similar to that in other centers (21). These results highlight the need for effective treatments with a rapid onset of action and few side-effects. Patients may reach PASS “Yes” without specific immunosuppressive drugs if they have a strong response to pyridostigmine or thymectomy, and also if spontaneous improvement or remission occurs in this autoimmune disorder.

Patients’ perceptions of their chronic illness change with time as they adapt to their disease disability, as demonstrated in disorders such as osteoarthritis (22, 23). Living with and adapting to chronic MG can gradually change a patient’s perception of disease consequences as they modify their lifestyle both actively and passively. Although the PASS “Yes” response is strongly related to an improvement in impairments as demonstrated on MGII and SSQ scales, patients also reach PASS “Yes” despite ongoing MG limitations with only a minority reaching complete remission, likely due to patient adaptation to their myasthenic state.

A weakness of our study is that it is a retrospective review, and future prospective studies are required to confirm these findings. Secondly, this study is exploratory in nature and the cohort of 84 patients is relatively small because our methods of assessment used novel scales developed in recent years and so the time interval for data collection started only after these scales were introduced in our clinic. However, these scales are complementary and assess multiple facets of the MG patient experience providing a comprehensive view of our patients’ experience. A future prospective trial study with a larger sample size will be able to address these limitations and further validate our findings. In addition, patients who reached a PASS “Yes” may change to PASS “No” during the course of their disease due to the fluctuating nature of MG or patient perception to disease or medication side effects. We lack this data, and we intend to address this in future studies.

## Conclusion

The PASS response shows that most patients with MG achieved satisfactory disease status within 25 months from diagnosis. Very-late-onset MG and those requiring only corticosteroid treatment had a significant shorter time to achieve a satisfactory symptom state.

## Data availability statement

The raw data supporting the conclusions of this article will be made available by the authors, without undue reservation.

## Ethics statement

The studies involving human participants were reviewed and approved by The University Health Network Research Ethics Board. Written informed consent for participation was not required for this study in accordance with the national legislation and the institutional requirements.

## Author contributions

RM-H, CB, and VB contributed to the manuscript concept, design, writing, and data interpretation of the study. RM-H organized the database and performed the statistical analysis. VB organized and provided guidance throughout the execution of the project. All authors contributed to the article and approved the submitted version.

## Conflict of interest

The authors declare that the research was conducted in the absence of any commercial or financial relationships that could be construed as a potential conflict of interest.

## Publisher’s note

All claims expressed in this article are solely those of the authors and do not necessarily represent those of their affiliated organizations, or those of the publisher, the editors and the reviewers. Any product that may be evaluated in this article, or claim that may be made by its manufacturer, is not guaranteed or endorsed by the publisher.

## Abbreviations

MGII: Myasthenia Gravis Impairment Index
SSQ: Single Simple Question
PASS: patient acceptable symptom state
RNS: repetitive nerve stimulation
SFEMG: single fiber EMG
P: prednisone
IST: non-steroidal immunosuppressant treatment.
